# The Relationship Between Reading Fluency and Arithmetic Fact Fluency and Their Shared Cognitive Skills: A Developmental Perspective

**DOI:** 10.3389/fpsyg.2019.01281

**Published:** 2019-05-31

**Authors:** Reut Balhinez, Shelley Shaul

**Affiliations:** Edmond J. Safra Brain Research Center, Department of Learning Disabilities, University of Haifa, Haifa, Israel

**Keywords:** reading fluency, arithmetic fact fluency, cognitive skills, rapid automatized naming, working memory, inhibition

## Abstract

This study investigated the underlying cognitive abilities which are related to both fluency in reading and arithmetic across different developmental phases of their acquisition. An unselected sample of children in first (*N* = 83), second (*N* = 66), and third (*N* = 67) grades completed several reading and arithmetic fluency tasks, as well as rapid automatized naming (RAN), working memory (WM), and inhibition measures. The results of a stepwise regression analysis revealed differences in the predictive models of fluency in both academic domains in first grade. However, similar patterns were found in the second and third grades. Specifically, in first grade reading fluency was predicted by inhibition and WM, while arithmetic fact fluency was predicted by RAN and WM. In contrast, in second grade both types of fluency were predicted by RAN and WM, and in third grade only RAN was found to be a predictor. Alongside the gradual reduction in the cognitive components participating in reading and arithmetic fluency, the results of the present study suggest that both fluencies share the same underlying cognitive mechanisms. Practical implications of the current results are discussed.

## Introduction

Although mathematical and language abilities are mostly considered two separate fields in the scientific literature and school curricula, there is strong scientific evidence for a close connection between them (for a meta-analysis: Singer and Strasser, 2017). According to a vast body of research, one of the most fundamental component, both in reading and arithmetic, is fluency. In today’s fast-paced society, the ability to read fluently and to perform basic calculations are essential abilities required for everyday life activities. The importance of these abilities is manifested in overall academic development and achievements (Price et al., 2013; Ruddy et al., 2018).

Most of the existing studies in the field of fluency investigated this construct and its developmental nature in two distinct disciplines: reading and arithmetic. A deep empirical examination revealed that only a small set of studies focused on fluency from a cross-domain perspective (e.g., Koponen et al., 2013). The majority of those studies referred to this construct as part of a wide range of skills (e.g., Van der Sluis et al., 2007). Furthermore, less is known concerning the developmental trajectories of the association between reading fluency and arithmetic fact fluency. The current study addresses these gaps by investigating the structure of fluency in both domains in a cross-sectional sample among first to third grade children.

Cross-domain studies have indicated that the association between reading and arithmetic is mainly derived from shared cognitive skills and processes (e.g., Durand et al., 2005; Blair and Razza, 2007; Best et al., 2011; Ashkenazi et al., 2013; Yeniad et al., 2013; Purpura and Schmitt, 2018). Although the role of the cognitive components has been examined in the study of both reading fluency (e.g., Warmington and Hulme, 2012) and arithmetic fact fluency (e.g., LeFevre et al., 2013), only a few studies focused on the overlap between them (e.g., Georgiou et al., 2013a, 2015). This study sought to expand the empirical knowledge regarding the contribution of varied cognitive components [i.e., working memory (WM), inhibition, and RAN] to the development of fluency through a cross-domain approach.

### Fluency in Reading and Arithmetic and Their Cross-Domain Connections

The term “fluency” was originally derived from the word “flow.” Traditionally, fluency is defined as the smooth and effortless production of speech and pronunciation (Chambers, 1997). In the last few decades, fluency received extensive attention in the fields of reading (e.g., Georgiou et al., 2008b) and arithmetic (e.g., Vanbinst et al., 2015).

Arithmetic fact, or number combinations, fluency refers to the automatic retrieval of simple single-digit facts from memory (Zaunmüller et al., 2009). This type of facts includes positive integers with the four basic math operations: addition, subtraction, multiplication, and division (e.g., 3 + 5 = 8, 4 × 6 = 24) (National Mathematics Advisory Panel, 2008; Vukovic et al., 2010). Fluency in arithmetic has been found to be an essential tool for overall mathematical achievements, especially during the years of elementary school (Nunes et al., 2012). Specifically, Fuchs et al. (2006) found that automatic retrieval of basic arithmetic facts predicts algorithmic computational skills and arithmetic word problem solving among third grade students. In this regard, Jordan et al. (2003) longitudinal study demonstrated that second graders with difficulties in arithmetic fluency were significantly more prone to low mathematics performance in later school years.

The development of mathematical fluency occurs in several stages, with different strategies used in each phase. In the early stage of acquisition, the young student relies mostly on “lower-level” strategies such as counting. At this point, individuals demonstrate and manipulate quantities using concrete representations (such as fingers and objects) and verbal counting (Geary et al., 1991; Crollen and Noël, 2015). As conceptual and procedural knowledge about numbers increases, “higher-order” strategies develop. In this relatively advanced stage, the student mentally decomposes a given arithmetic problem into an easy and familiar sub-computation equation [for example, the math problem 8 + 5 might be decomposed to (5 + 5 = 10) + 3 = 13] (Laski et al., 2013). The efficient use of counting and decomposition strategies through repeated practice, gradually strengthens the association between the problem and its correct answer in WM. Memory-based retrieval of facts occurs when this association becomes established as a declarative information in long-term memory (Geary et al., 1991). At this phase, arithmetic facts retrieval from memory is more efficient, as reflected by higher accuracy and lower rate of performance in which the problem is solved and automatic retrieval is used (Mabbott and Bisanz, 2003). According to the Mathematical education curriculum for elementary school in Israel Office of Pedagogical Affairs, Israel Ministry of Education (2006), students at the end of the third grade gradually shift to using automatic retrieval of basic arithmetic facts of integers numbers in all four operations together with other strategies. Therefore, the examination of students from first through third grade might provide the full developmental range of arithmetic fact fluency during the initial stages of learning through the mastery recall of factual knowledge.

Reading fluency, in its functional definition, is described as the ability to perform oral reading as rapidly and accurately as possible (LaBerge and Samuels, 1974; Meyer and Felton, 1999; Wolf and Katzir-Cohen, 2001; Hudson et al., 2005). Mastery of reading fluency has a main role in the manifestation of higher order processes, such as reading comprehension (e.g., Fuchs et al., 2001; Klauda and Guthrie, 2008; Kim et al., 2010). In general, reading acquisition initially relies on the decoding of written graphemes into phonemes. Gradually, as sub-lexical identification is established, the reader becomes capable of automatically processing larger orthographic units, such as a whole word, while relying on lexical access. Specifically, it has been suggested that reading fluency is a multi-componential structure which entails the integration of various subskills (LaBerge and Samuels, 1974; Wolf and Katzir-Cohen, 2001). Rapid and efficient word recognition requires practice through repetitive exposures (Logan, 1988).

However, considerable differences were found in the development of reading fluency across orthographies (e.g., in Arabic: Saiegh-Haddad, 2005; in German: Landerl and Wimmer, 2008; in Finnish, Greek, and English: Georgiou et al., 2012; in English and Hebrew: Katzir et al., 2012). Specifically, in the case of Hebrew, young children learn to read with the pointed script, which is considered a consistent “shallow” orthography. The high transparency of written Hebrew contributes to the rapid growth in word reading fluency during the first grade at school (Lipka et al., 2016). In fourth grade, the transition to the un-pointed script occurs and simultaneously the rapid mastery of fluency slows (Share and Bar-On, 2017). In addition, at this phase reading fluency involves with different “higher-order” linguistic processes (Katzir et al., 2012). In order to limit the effect of these factors, the present study focused on the development of word reading fluency through the pointed Hebrew script in which the students mostly exposed to during the first 3 years of elementary education.

The development of reading fluency in the first stages is similar to the mathematical fluency development. The children enter school with basic letter and their sounds knowledge, and start to establish the association between them in the next stage as words level knowledge starts to build. In the last stage word presentations are created in the long-term memory on which the fluency develops (Fuchs et al., 2016). Due to the similarities between the first stages of the development of reading and mathematical fluency which involve the common processes of establishment of strong associations which are stored in the long term memory, it is likely that the same brain and cognitive mechanizes are involved in both domains (Qin et al., 2014).

Although fluency in reading and in arithmetic are referred to as distinct abilities, there is scientific evidence for a direct connection between them. Hence, significant correlations were found among the two fluencies in second, third (Koponen et al., 2016), and fourth graders (Koponen et al., 2007). Another indication of the inseparable link between fluent reading and arithmetic fact retrieval is further supported by studies which examined children with learning disabilities compared to typically developing controls. Accordingly, Mammarella et al. (2013) argued that reading disability (i.e., dyslexia) has a negative influence on arithmetic fact retrieval. Moreover, a majority of the children with arithmetic deficits (i.e., developmental dyscalculia) also demonstrate reading difficulties (Von Aster and Shalev, 2007). In this regard, it is important to mention that most of the studies concerning the cross-domain association, focused on learners with difficulties and disabilities. In addition, systematic examination of the connections between these abilities across different developmental phases has yet to be fully studied.

The unique association between fluency in reading and arithmetic can be explained by two different approaches. First, from a heritability perception, substantial genetic overlap was found in timed measures, including fluency, in both academic domains. For example, in the longitudinal twin study of Hart et al. (2010), the researchers found a moderate relationship between arithmetic fact retrieval and word reading fluency (*r* = 0.43). Another related research by Petrill et al. (2012) pointed to a stable correlation between these abilities across first (*r* = 0.41 at age 10) and second (*r* = 0.40 at age 11) measurement occasions. A second explanation of the unique cross-domain overlap between these abilities is that fluency, in both domains, relies on the same cognitive mechanisms. The current study adopted the cognitive approach as a possible explanation for the overlap between fluency in the two academic domains.

### Cognitive Components in Reading and Arithmetic Fact Fluency

Many cognitive processes seem to be involved in reading and arithmetic skills, and many of these processes are similar in both domains due to the comparable development of these abilities in their first stages. However, the present study sought to assess the relative contribution of three theoretically noticeable predictors including rapid naming, WM and inhibitory control, to the development of fluency in reading and arithmetic.

Rapid automatized naming (RAN) is defined as the ability to name a stimulus, as fast and accurate as possible, (such as letters, digits, colors or objects) (Koponen et al., 2013). This relatively fundamental skill appears to represent the most basic mechanism of fluency in terms of fast and accurate serial processing of symbols while reading (i.e., strings of letters) and calculating (i.e., combination of digits and operation marks). Therefore, it is not surprising that RAN was found to be related to both reading fluency and arithmetic fact fluency.

The association between RAN and reading fluency across different orthographies has been well-documented over the past decade (e.g., Georgiou et al., 2009, 2013a; see also Araújo et al., 2015 for evidence from a meta-analysis). Generally, alphanumeric RAN tasks (digits and letters) are more strongly associated with reading performance than non-alphanumeric RAN (objects and colors) (Bowey, 2005; Georgiou et al., 2009; Araújo et al., 2015). In this regard, prior studies indicated that the predictive role of RAN tasks varies across orthographies. Specifically, RAN digit was found as a stronger predictor of word reading fluency in transparent orthographies such as Hebrew (Shechter et al., 2018) and Greek (Georgiou et al., 2008b).

More recently, however, there is a growing interest in the relationship between RAN and arithmetic fact fluency. Accordingly, several studies have indicated that RAN is significantly associated with children’s efficiency in basic arithmetic calculation from kindergarten (Cui et al., 2017) through the first years of formal instruction in elementary school (Berg, 2008). The majority of studies which employed a wide range of RAN tasks reported that number-specific RAN measures (such as digit) were more strongly related to arithmetic fact fluency (e.g., Swanson and Kim, 2007; Hornung et al., 2017).

The few cross-domain studies conducted on rapid serial naming have reported that RAN was a significant predictor of fluency both in reading and arithmetic (Georgiou et al., 2013b; Koponen et al., 2013, 2016). Likewise, RAN was also a predictor which accounted for the covariance between the two fluencies (Koponen et al., 2007; Korpipää et al., 2017). These results reinforce the assumption that fluency in reading and arithmetic share similar underlying processes, that are based on the efficient process of familiar representation (Van der Sluis et al., 2007). Despite the acknowledged contribution of rapid naming to fluency performance, only few studies have examined the relationship between RAN and fluency in reading and arithmetic concurrently. Thus, the current investigation aimed to expands the existing knowledge on the shared aspect of rapid-naming to the development of fluency in both academic domains from a cross-domain perspective. Whereas the findings concerning the role of RAN in both reading and arithmetic fluency are consistent, the scientific literature about the role of other cognitive components, is less clear.

Beyond the necessity of rudimentary visual mapping of the presented stimuli, fluency performance demands higher-order cognitive abilities. These processes are typically referred to as executive functions (EFs), which are responsible for self-regulation and goal-oriented behavior (Monette et al., 2011). According to the structural model suggested by Miyake et al. (2000), the three main subcomponent skills of EFs are WM, inhibition, and cognitive flexibility. In spite of these well-founded multi-componential framework, the present study focused on WM and inhibitory control. WM refers to the ability to temporarily store and manipulate information (Baddeley, 1992). Inhibition involves the ability to suspend an automatic response in order to select more compatible behavior (Dowsett and Livesey, 2000). The selection of these two EF’s constructs derived from several fundamental reasons.

From a developmental perspective, the diversity between EF’s constructs appears to be highly affected by age-related changes of these skills. Thus, in contrast to the significant differentiation between these three basic functions among young adults (Miyake et al., 2000), it appears that EF’s components tend to be unitary during the early childhood years (Zelazo and Müller, 2002). However, there is some evidence which supports primary and unique diversity between WM and inhibition factors among preschoolers (Miller et al., 2012) and elementary school age children (Brocki et al., 2010). Moreover, in these studies’ cognitive flexibility (or shifting) was not found as a distinct component of EF’s.

The developmental nature of WM and inhibition is further manifested in the interaction between them (Hasher and Zacks, 1988; Hasher et al., 1999; Gazzaley et al., 2005). Empirical studies revealed that children’s inhibitory control develops earlier than the other EF’s processes (Senn et al., 2004) and supports the performance of the other executive skills (Barkley, 1997). Specifically, inhibitory control plays a main role in restraining, limiting, and deleting irrelevant information from WM capacity (Hasher and Zacks, 1988; Conway and Engle, 1994; Hasher et al., 1999).

The interaction between these executive skills is further evidenced in the context of academic performance. For example, Monette et al. (2011) found that inhibitory control was related to mathematics achievements only when WM measures were omitted from the analyses. Moreover, a substantial series of studies was conducted on the role of WM and inhibition have found significant and consistent relationship between these particular EF’s skills in a wide range of reading skills (e.g., Nevo and Bar-Kochva, 2015; Cummine et al., 2018) and mathematics (e.g., Friso-van den Bos et al., 2013; Gilmore et al., 2015). In contrast, cognitive flexibility did not uniquely predict academic performance (Espy et al., 2004; Van der Sluis et al., 2007).

The relationship between WM and mathematics has been well documented in the early primary grades (De Smedt et al., 2009; Meyer et al., 2010). Relatively many studies assessed the direct effect of WM on simple arithmetic fluency (e.g., Adams and Hitch, 1997; Hecht, 2002; Kaufmann et al., 2004; Soltanlou et al., 2015). In one such study, Adams and Hitch (1997) found that WM capacity has a main role in simple addition problem solving efficiency among children. WM primarily involves the execution of procedural strategies, such as counting and decomposition, while solving arithmetic problems (Adams and Hitch, 1997; Hecht, 2002). For example, a substantial indication for the connection between WM capacity and strategy usage was found in a study which examined children with mathematical disabilities compared to their typical development peers (Geary et al., 2004). Their results revealed that children with low WM capacity tended to rely on less sophisticated strategies (such as finger counting). Conversely, children with high WM capacity used more sophisticated and mature strategies (such as decomposition). High WM ability was also linked to effective shifting from rudimentary strategies to more advanced strategies. Geary et al. (2004) also found that WM was no longer related to arithmetic fact fluency in third and fifth grade. Accordingly, it seems that the reduced importance of WM is associated with the attainment of automaticity in the retrieval of arithmetic facts from long term memory. However, less is known about the developmental role of WM in arithmetic fluency across the lower grades of elementary school.

In the initial phases of reading acquisition, WM enables the segmentation of graphemes into phonemes. The relevant information is simultaneously stored and held in mind while integrated into a single word (Rohl and Pratt, 1995). In view of that, some studies suggested that WM has a unique contribution to children’s reading fluency development (Swanson and Jerman, 2007; Berninger et al., 2010; Jacobson et al., 2011). For example, Berninger et al. (2010) found that WM predicted reading fluency in second, fourth, and sixth grade. However, most of the research on the association between WM and reading is dominated by an emphasis on the accuracy variable in word identification (e.g., Rohl and Pratt, 1995; Swanson and Jerman, 2007; Georgiou et al., 2008a). As a result, less is known about the contribution of WM capacity to fluent reading, especially across different developmental phases.

Since arithmetic facts are stored in associative memory networks, inhibition might be needed in the suppression of an incorrect, yet related, solution for an arithmetic problem, for example, suppressing the answer 8 for the number combination 4 × 4 (Campbell et al., 2011; Cragg and Gilmore, 2014; Cragg et al., 2017). Despite this notion, findings regarding the relationship between inhibition and single-digit arithmetic facts are somewhat more variable. Furthermore, the available literature has been collected based on samples of young adults and beyond. For example, in a recent study by Gilmore et al. (2015) no relationship emerged between inhibition and arithmetic factual knowledge in adolescents and young adults. Conversely, several authors found close connections between these skills among samples of adults (Campbell, 1990; Barrouillet et al., 1997). These contradictory findings might be derived from different methods for arithmetic fact fluency measurement. While some assessed fact mastery through a verbal response to a given problem (Gilmore et al., 2015), others evaluated facts retrieval by recognition of the correct answer when presented along with distractors (Campbell, 1990; Barrouillet et al., 1997). Since different responses are required, it might be assumed that each task stimulates the inhibitory system in a different manner (Noreen and MacLeod, 2015).

In reading, Blair and Razza (2007) assumed that inhibition facilitate the processing of relevant information during the segmentation of graphemes into phonemes. Furthermore, Conners (2009) claimed that inhibitory control is mostly required when a word is deciphered incorrectly. Experimental indication of the association between inhibition and reading fluency was intensively evidenced by the Stroop interference task. In the classic color-word Stroop task (Stroop, 1935) participants are required to rapidly name the color ink of the printed word while ignoring and inhibiting the word reading (for example, the word “BLUE” presented in red ink). The Stroop effect premise is that a skilled reader will be slowed and experience more interference by the incongruous word-color condition. Indeed, increasing Stroop interference was found among readers in first grade (Stanovich et al., 1981) and continues to increase in children classified as fourth grade reading level (Schadler and Thissen, 1981). However, many studies documented age-related changes in the level of interference due to the development of the inhibitory mechanism (e.g., Dempster, 1992; Leon-Carrion et al., 2004). Thus, Pritchard and Neumann (2004) reported a significant gradual reduction in the level of interference in children around the age of 10 years. Therefore, it is not surprising that a high level of interference has been found to be associated with low performance in word reading fluency in the third grade (Schwanenflugel et al., 2006). However, the reviewed literature revealed that little attention has been paid to the predictive role of inhibitory control in the development of reading fluency during the early years of elementary school.

In conclusion, despite the critical importance of RAN, WM and inhibition in academic achievements and their dramatic growth during the first years of elementary school, most of the present literature examined individual cognitive component with regards to fluency in a specific domain (reading or arithmetic) at a certain developmental stage. As a result, the concurrent predictive role of these cognitive skills in the acquisition of fluency, in reading and arithmetic, at different developmental phases is still unclear. Such theoretical understanding concerning the underpinning cognitive factor of fluency might provide practical and pedagogical tools to support the one of the most basic learning skills in reading and mathematics.

### The Current Study

Considering the reviewed literature, only a few studies have been conducted on the cross-domain association between fluency in reading and arithmetic across different developmental phases of the acquisition. In addition, further examination is required concerning the predictive power of varied cognitive components (RAN, WM, and inhibition), measured concurrently, in the development of fluency in the two academic domains. The current study also aimed to reveal the shared cognitive components of fluency in reading and arithmetic, which might shed light on the cross-domain connection between them.

This study addressed the following two research questions:

(1) What are the connections between reading and arithmetic fact fluency among children at different stages of development, from the first grade till the third grade, and do the connections remain the same or change with the development of these abilities? Drawing on evidence from previous work (Koponen et al., 2007, 2016), we expected to find a systematic correlation among these abilities in first, second, and third grades.
(2) Which cognitive components (RAN, WM, and inhibition) are related to reading and arithmetic fact fluency in each developmental stage and what are their shared cognitive predictors during the first years of elementary school? As the RAN measure proved to be a strong predictor of fluency in reading and arithmetic, we expected these finding to be replicated from first through third grade. In consideration to the interplay between WM and inhibition along with the mixed evidence regarding their predictive role in the development of both types of fluency, no clear hypothesis can be made. As a result, uncertainty also arises concerning fluencies shared cognitive components. However, in light of the notion that less skilled learners rely more on cognitive resources compared to more proficient students (Bull and Scerif, 2001), it can be expected to find a gradual reduction in the role of the cognitive components as the development of reading and arithmetic fluency progressed.

## Materials and Methods

### Participants

The participants were 216 Hebrew-speaking children recruited from two local elementary schools in the north of Israel from different socioeconomic backgrounds: medium-low (*N* = 133) and medium-high (*N* = 83). Sixty-seven first graders (mean age = 6 years 5 months, *SD* = 0.47; 35 boys, 32 girls), 83 second graders (mean age = 7 years 7 months, *SD* = 0.50; 41 boys, 42 girls), and 66 third graders (mean age = 8 years 6 months, *SD* = 0.49; 35 boys, 31 girls). For this study, participants were excluded if they did not complete the test battery or could not read independently. All participants were Hebrew typical developing children with no neurological disorders. Parental written consent was given to 85 children in each grade prior to task administration. The attrition rate varied from grade to grade and was 22.2% (*n* = 67) in first grade, 2.4% (*n* = 83) in second grade, and 22.3% (*n* = 66) in third grade.

### Measures

#### Rapid Automatized Naming (RAN)

##### Rapid automatized naming of digits (Shany et al., 2006)

The RAN digits’ subtest assesses the ability to name a series of 50 written stimuli as rapidly as possible. The test includes five digits (1, 5, 9, 3, 7), each repeated randomly 10 times. The reliability of these task is 0.74 in second grade and 0.69 in fourth grade.

#### Executive Function Measures

##### Working memory

Two tasks were used to measure verbal WM: The children’s size-ordering task (CSOT; McInerney et al., 2005) and the digit span backward (WISC-III, Wechsler, 1991), which require simultaneous storage and manipulation of verbal information.

The CSOT (McInerney et al., 2005) contains a list of common objects (e.g., window, book) read aloud at a rate of one item per second. Children were required to reproduce them verbally in order of size, from smallest to largest (e.g., book, window). The test comprises seven blocks with two items per block. The level of difficulty increased gradually, beginning with a length of two items to a maximum length of seven items. The test was discontinued when two mistakes occurred in the same block. Granvald and Marciszko (2016) reported reliability of CSOT is 0.63.

Digit span backward (WISC-III, Wechsler, 1991): Children were verbally presented with a sequence of random numbers and asked to recall them in the reverse order, from the last number they had heard to the first one. The test was discontinued when two mistakes occurred in the same block. The measure used was the total number of correct items recalled.

##### Inhibition (Stroop, 1935)

The Color–Word Interference test consists of three conditions: reading words (i.e., neutral condition), naming color patches (i.e., congruent condition), and naming the color in which words are printed instead of reading the words, for example, the word RED presented in green print (i.e., incongruent condition). Each condition contains 65 printed items presented in five rows of 13 items each. Participants were asked to respond to the pertinent stimuli (i.e., word or color) as quickly and accurately as possible within 45 s. The measure used was the received magnitude difference between the neutral condition and the incongruent condition. Prior research reported that the temporal reliability of the three conditions is *r* > 0.80 (Connor et al., 1988; Sacks et al., 1991; Graf et al., 1995).

#### Word Reading Fluency Measure

##### Single-word reading speed test (Schiff et al., 2006; adapted from the TOWRE test, Torgesen et al., 1999)

The test of oral reading accuracy and fluency contains a list of 104 fully vowelized words increasing in difficulty and varying in frequency, length, phonological and morphological structure. Children are requested to read the words aloud as quickly and accurately as possible. The measure used was the number of correct words read per 45 s. Cronbach’s alpha for second grade is 0.90.

#### Arithmetic Fact-Fluency Measure

##### The WJ-III math fluency test (Woodcock et al., 2001)

Children were instructed to complete as many simple single-digit addition, subtraction, and multiplication facts within 3 min, skipping those they do not know. The measure used was the number of problems solved correctly within 3 min. The reported reliability is between 0.90 and 0.93.

### Procedure

The tests described earlier were administered as part of a larger test battery within an extensive longitudinal intervention study conducted in Israel. The data was collected prior to implementation of the intervention program. Participants were tested individually in a quiet room at the children’s school, during school hours, by trained examiners. The tests were administered in random order. Short breaks were allowed as needed by the child between tests.

### Statistical Analysis

Table 1 shows the means, standard scores, and one-way ANOVA for the three grades on fluency (in reading and arithmetic), cognitive measures (WM and inhibition), and RAN. The univariate tests revealed a significant main effect for grade across all measures, with consistent improvement from first to third grade. Due to the group’s adequately differentiated verification, the three experimental grades in the study were considered separately.

**Table 1:** Descriptive statistics and one-way ANOVA among tasks in first (*N* = 67), second (*N* = 83) and third (*N* = 66) grades.

	First grade		Second grade		Third grade			
Measures	*M*	*SD*	*M*	*SD*	*M*	*SD*	*F*	${\text{η}}_{\text{p}}^{2}$
Word reading fluency – TOWRE (correct words per 45 s)	29.35	10.84	48.83	13.61	58.78	16.74	78.81^∗∗^	0.42
Arithmetic fact fluency (correct answers per 3 min)	32.22	11.29	42.41	14.03	54.71	14.46	47.75^∗∗^	0.30
RAN digits (time)	44.82	10.71	35.95	8.22	31.35	7.88	39.52^∗∗^	0.26
Digit span backward (total correct items)	3.00	0.88	3.67	1.12	4.00	1.35	13.65^∗∗^	0.11
WM – CSOT (total correct items)	4.01	1.26	4.54	1.42	4.91	1.34	7.48^∗^	0.06
Inhibition – Stroop (total correct items per 45 s)	20.41	16.56	37.88	7.85	38.01	9.97	51.96^∗∗^	0.32

*^∗^p < 0.05; ^∗∗^p < 0.01.*

Then a correlation analysis was performed across all tasks in order to examine (1) the developmental nature of the connection between fluency in reading and arithmetic; (2) the relationship between fluency, in both domains, and cognitive components across different developmental phases. Finally, a stepwise multiple regression was conducted to evaluate which cognitive skills would predict fluency in reading and arithmetic at early and advanced phases of acquisition. Fluency in reading and arithmetic were concluded as dependent variables and the EF measures of verbal WM (CSOT and digit span backward), inhibition (Stroop), and RAN digits as potential predictors.

## Results

### Pearson’s Correlation Analyses

The correlation between reading fluency and arithmetic fact fluency with RAN and EF variables for first, second, and third grades are presented in Table 2. Inspection of the correlation table reveals consistent moderate correlations between arithmetic fact fluency and word reading fluency in all three grades (*r* = 0.54 in first grade, *r* = 0.46 in second grade, and *r* = 0.51 in third grade).

**Table 2:** Correlations between reading fluency, arithmetic fact fluency, and cognitive measures for first (*N* = 67), second (*N* = 83), and third (*N* = 66) grades.

	First grade					Second grade					Third grade				
Variable	1	2	3	4	5	1	2	3	4	5	1	2	3	4	5
1. Word reading fluency – TOWRE															
2. Arithmetic fact fluency	0.54^**^					0.46^**^					0.51^**^				
3. RAN digits	-0.49^**^	-0.55^**^				-0.42^**^	-0.51^**^				-0.54^**^	-0.40^**^			
4. WM – Digit span backward	-0.16	0.12	-0.27^*^			0.27^*^	0.30^**^	-0.11			0.20	0.14	-0.26^*^		
5. WM – CSOT	0.34^**^	0.26^*^	-0.15	0.10		0.32^**^	0.23^*^	-0.39^**^	0.34^**^		0.17	0.04	-0.15	0.31^*^	
6. Inhibition – Stroop	0.60^**^	0.32^**^	-0.55^**^	0.16	0.00	0.26^*^	0.25^*^	-0.21	0.14	0.08	-0.02	-0.01	-0.05	-0.07	0.08

*^∗^p < 0.05; ^∗∗^p < 0.01.*

Unsurprisingly, the RAN measure was significantly correlated with both reading fluency and arithmetic fact fluency across all three grades. The EF components did not show the same cross-grades consistency. Thus, reading fluency and arithmetic fact fluency were correlated with EFs in first and second grades but not in third grade.

Interestingly, the correlations within first, second, and third grades are similar. Specifically, in first grade reading fluency and arithmetic fact fluency were both correlated with WM (CSOT) and inhibition (Stroop). In second grade, however, fluency in reading and arithmetic were significantly related with both WM tests (CSOT and digit span) and inhibition (Stroop). In third grade, fluency measures were correlated solely with the RAN.

### Multiple Regression Analyses

A statistics regression model for fluency in reading and arithmetic as dependent variables, and cognitive components (RAN, WM, and inhibition) as predictors is provided in Table 3. The predictor measures were entered simultaneously with a stepwise function. This approach allowed us to identify the best predictive variables for fluency as a function of grade according to their degree of contribution.

**Table 3:** Stepwise regression analysis: best model to predict reading fluency and arithmetic fact-fluency in first (*N* = 67), second (*N* = 83), and third (*N* = 66) grades.

		Model				
	Variables	*R*^2^	*F*	*R*^2^ change	*F* change	β
First grade (*n* = 67)	Reading fluency					
	Inhibition – Stroop	0.36	36.36	0.36	36.36^∗∗^	0.56
	WM – CSOT	0.47	28.22	0.11	13.30^∗∗^	0.33
	Arithmetic fact fluency					
	RAN digits	0.28	26.20	0.28	26.20^∗∗^	-0.51
	WM – CSOT	0.33	16.06	0.05	4.52^∗^	0.22
Second grade (*n* = 83)	Reading fluency					
	RAN digits	0.16	17.44	0.17	17.44^∗∗^	-0.39
	WM – Digit span backward	0.22	11.81	0.05	5.28^∗^	0.23
	Arithmetic fact fluency					
	RAN digits	0.26	29.64	0.26	29.64^∗∗^	-0.49
	WM – Digit span backward	0.33	19.70	0.06	7.45^∗^	0.25
Third grade (*n* = 66)	Reading fluency					
	RAN digits	0.30	28.45	0.30	28.45^∗∗^	-0.55
	Arithmetic fact fluency					
	RAN digits	0.17	13.63	0.17	13.63^∗∗^	-0.42

*^∗^*p* < 0.05; ^∗∗^*p* < 0.01.*

For word reading fluency in first grade, inhibition uniquely explained 36% of the variance, whereas WM (CSOT) contributed an additional 11% of the variance. The two variables collectively explained approximately 47% (adjusted *R*^2^ = 0.45, *p* < 0.01) of the variance in reading fluency. In second grade, RAN explained 16% of the variance and verbal WM added to the explanation an additional 5% of the variance. The two variables collectively explained approximately 22% (adjusted *R*^2^ = 0.21, *p* < 0.05) of the variance. In third grade, only RAN was found to be a significant predictor, with 30% (adjusted *R*^2^ = 0.29, *p* < 0.001) of unique variance in word reading fluency.

For arithmetic fact fluency in first grade, inhibition uniquely explained 28% of the variance whereas WM (CSOT) contributed an additional 5% of the variance. The two variables collectively explained approximately 33% (adjusted *R*^2^ = 0.31, *p* < 0.05) of the variance in arithmetic fluency. In second grade, RAN explained 16% of the variance and verbal WM added to the explanation an additional 6% of the variance. The two variables collectively explained approximately 22% (adjusted *R*^2^ = 0.31, *p* < 0.01) of the variance. In third grade, only RAN was found to be a significant predictor, with 17% (adjusted *R*^2^ = 0.16, *p* < 0.001) of unique variance in arithmetic fact fluency.

Consequently, RAN was found to be a constant predictor of arithmetic fluency across all grades. In addition, a comparison between the predictive models of reading and arithmetic fluency in each class revealed an interesting trend. While second and third grade fluency in reading and arithmetic were significantly predicted by the same components, first grade displayed different patterns.

## Discussion

The present study aimed to explain the cross-domain overlap between reading and arithmetic fact fluency through a deep examination of their shared underlying cognitive factors (RAN, WM, and inhibition). This study is among the few to investigate this topic among an unselected sample of children across the lower elementary grades.

As hypothesized, the correlation analysis revealed a moderate, yet consistent, cross-domain association between fluency in reading and arithmetic from first grade to third grade. This is in line with previous studies linking fluency in reading and arithmetic in the early years of elementary school (Koponen et al., 2007, 2016). In addition, these results have also been confirmed by twin studies which verified the possibility of a common fluency component associated with a shared genetic factor (Hart et al., 2010; Petrill et al., 2012). It is important to note that the connection between the two fluencies was found at all stages of development from the first stage of acquisition until the end of third grade in which the children have fully acquired their fluency in both domains.

There can be two possible explanations for these findings. First, it may be suggested that these connections might derive from similarities in the course of acquisition of these abilities (Kulak, 1993). In the initial phases of development, both academic skills rely on serial processing of written symbols, which represent concrete information. Specifically, reading involves sequential decoding of graphemes to phonemes while arithmetic is based on the representation of a given number quantity through serial counting. Gradually, as the strategies become more efficient and sophisticated, the ability to process larger units in reading (i.e., words) and calculations (i.e., arithmetic facts) develops. Fluency mastery, in both academic domains, is assumed to rely mainly on automatic retrieval from memory, achieved through repeated exposure and continuous practice (Logan, 1988; Kulak, 1993). In this context, the results of the present study revealed significant variance in the performance of fluency in reading and arithmetic, which improved across the three grades examined. These developmental trends might be related to the extensive exposure, practice, and experience in reading and arithmetic calculation during the first years of formal education. Gradually, as learning progresses, the young student specializes in each domain studied and becomes more fluent.

A second explanation for the cross-domain overlap between reading and arithmetic has been linked to shared cognitive processes (e.g., Koponen et al., 2007; Berg, 2008; Simmons and Singleton, 2008; Hart et al., 2009; Dickinson and Porche, 2011; Brunswick et al., 2012). In view of this approach, the next section will address the unique role of the cognitive components (RAN, WM, and inhibition) in the development of fluency, in both academic domains, across the three initial years of elementary school.

### Shared Cognitive Predictors of Reading and Arithmetic Fact Fluency

The predictive models obtained in the present study, revealed an interesting developmental pattern concerning the shared cognitive predictors of fluency in both academic domains in first, second, and third grades.

In first grade, similarities and differences were found in the cognitive predictors of fluency in reading and arithmetic. Thus, the strongest predictor of reading fluency was inhibition, while RAN explained most of the variance in the ability to perform arithmetic calculations.

The fact that inhibitory control was found to be a predictor of reading fluency but not of arithmetic fact fluency might be related to differences in their processes and the particular type of knowledge they tap. Automatic reading is a complex ability which entails the synchronization of many components and information (LaBerge and Samuels, 1974) such as semantic, syntactic, and morphological processes (Wolf and Katzir-Cohen, 2001). Efficient word retrieval is established through repeated exposure to orthographic representations (Logan, 1988; Share, 1995). In the initial course of reading acquisition, the novice reader frequently encounters unfamiliar words which might evoke relevant and irrelevant information. In this case, inhibitory control might be essential for the suppression of the irrelevant information in favor of the relevant information. First grade arithmetic curricula, however, focus on the ability to perform addition and subtraction calculations only within 20 and base 10 numbers. Thus, the limited involvement of the inhibitory system in arithmetic fact fluency might reflect low levels of “competition” between the correct answer (relevant information) and other incorrect solutions (irrelevant information) and may also be due to the fact that is it an easier task which involves less information as opposed to reading in the initial stages.

Unexpectedly, the results of the present study revealed that RAN contributed significantly to arithmetic fact fluency but not to reading fluency in first grade. These findings are inconsistent with previous studies concerning the cross-domain predictive role of RAN in both types of fluency (Georgiou et al., 2013b; Koponen et al., 2013, 2016) and contrary to the strong evidence for the significant relationship between RAN and reading fluency (e.g., Araújo et al., 2015). This surprising finding can be explained by the fact that most of the existing studies about the cognitive predictors of reading fluency did not include tasks that assess inhibition concurrently with RAN. It may be that the inclusion of the inhibition task removed the contribution of the RAN to reading fluency due to the major role of inhibition in the first stages of reading acquisition as mentioned above. The retrieval of the correct stimulus in the RAN may also involve inhibition of competing stimuli which demands inhibition as well as other processes which are involved in naming, thus the addition of a pure inhibition task removed the contribution of the RAN.

Interestingly, in the present study WM (measured by the CSOT task) was found to be a common predictor of first graders’ reading and arithmetic fluency. This finding is in line with research showing the same predictive relations between WM and fluency in reading (e.g., Berninger et al., 2010) and arithmetic (e.g., Soltanlou et al., 2015). At this age, it is likely that reading and arithmetic are not yet completely fluent. Presumably, the young learners use different procedures which rely particularly on WM capacity.

Specifically, it can be assumed that WM is involved in grapho-phonemic decoding processes in reading, and strategy usage in simple arithmetic calculation. In this context, the specific inclusion of the CSOT task in the predictive models, might provide a closer observation of the role of WM capacity not only in reading and arithmetic but also in their fluency. The CSOT task entails complex manipulation of information simultaneously through verbal and visual processing in order to rearrange familiar objects according to their size (McInerney et al., 2005). This multi-step processing might emphasize, even more, the great involvement of WM processing in the early developmental stages of fluency acquisition. In addition, it can be assumed that fluency, in reading and arithmetic, might involve verbal and visual processing. In support of this, Georgiou et al. (2015) found that visual-spatial WM play a significant role in arithmetic fact fluency at the end of the first year of elementary school, even after controlling for other cognitive predictors. In contrast spatial WM contributed to reading fluency only when entered first to the model (Georgiou et al., 2015).

Furthermore, the simultaneous contribution of WM and inhibition to fluent reading might indicate the high cognitive load required for this ability in first grade. This finding is also reinforced by previous evidence as to the interaction between these cognitive mechanisms (e.g., Gazzaley et al., 2005). In arithmetic fact fluency it can be assumed that the increasing load of WM capacity might be related to greater usage of less sophisticated concrete strategies such as counting. These strategies might demand maintenance and manipulation of quantities through a mental visual imagery in the WM (de Hevia et al., 2008).

Alongside the differences in the cognitive components involved in fluency in first grade, similar predictive patterns were found in second and third grades. As initially hypothesized, RAN digits had the strongest contribution to fluency performance by the end of the second year of elementary school. In accordance with the results obtained in the present study, a substantial body of research in reading and mathematics links RAN to fluency (for a meta-analysis: Araújo et al., 2015; Koponen et al., 2017). A recent longitudinal study by Koponen et al. (2013, 2016) revealed the powerful predictive relations of RAN with fluent mastery of reading and arithmetic. Robust findings with regards to the association between RAN and fluency were also found among young children with difficulties in reading (Meyer et al., 1998; Shany and Share, 2011) and arithmetic (Mazzocco and Grimm, 2013). A possible explanation of these findings is that both fluency and RAN tap the same mechanism by which visual-verbal stimuli, such as digits and letter strings, are efficiently stored in and retrieved from long-term memory (Koponen et al., 2007).

Another common cognitive predictor of fluency in reading and arithmetic among second graders is WM (measured by the digit span backward task). The digit span tasks were widely classified as a measure of the phonological loop subsystem which entails the manipulation, retention, and articulatory processing of verbal information (Baddeley, 2003). In this context, previous findings demonstrated the importance of the phonological WM for the development of reading (Gathercole and Baddeley, 1993) and arithmetic calculation (Seitz and Schumann-Hengsteler, 2002).

As in first grade, the involvement of WM resources might indicate that these skills are not fully fluent at this stage of acquisition. Yet, a comparison between first and second grade might point to a developmental change in WM aspects involved in fluency. This assumption derives from the fact that fluency, in both academic domains, was predicted by different WM tasks in first (CSOT) and second grade (digit span backward). Similar to the CSOT task, there is growing evidence that the digit span backward task entails both visual imagery and verbal processes (Hoshi et al., 2000). However, these tasks differ substantively in their properties, demands, and in level of complexity. In contrast to the CSOT task, the digit span backward task seems to incorporate fewer procedures. Specifically, the list of items in the CSOT task must be completely rearranged by the size of the objects, while the digit span entails the recall of a sequence of digits in the reverse order. Moreover, digit span tasks were widely classified as a measure of the phonological loop subsystem which entails the processing of verbal information (Baddeley, 2003). Therefore, compared to the CSOT task it seems that the manipulation in the digit span backward task requires a lower WM load. Thus, although it appears that second graders still tend to use strategies while reading and calculating, it can be assumed that they select more sophisticated procedures and execute them more efficiently.

As hypothesized, a reduction was found in the cognitive components participating in fluent reading and simple arithmetic calculation in third grade. At this phase, fluency in both academic domains was predicted only by RAN. These results might indicate that the orthographic representation of the written word and the solution for the arithmetic problem are mostly retrieved directly from memory. As fluency in reading and arithmetic is more established, the cognitive resources are probably directed to higher order processes such as reading comprehension and arithmetic multi-step problem solving.

These cross-sectional data provide evidence for a developmental change in the cognitive predictors of fluency in reading and arithmetic from first through third grade. As the predictive models of fluency in both academic domains were different in first grade, the two fluencies were predicted by the same cognitive components in second and third grades. Overall, the results of the present study suggest that reading and arithmetic fact fluency share the same underlying cognitive mechanisms. These findings might shed light on the cross-domain connection between fluency in reading and arithmetic, considered one of the most fundamental abilities in the primary grades. Furthermore, this study emphasized the importance of strengthening basic cognitive skills which will help improve fluency in both domains from early childhood, in order to facilitate the entrance to school and the beginning of the academic learning. In addition, the connection between these two types of fluency and the overlap between them which may lead to the influence of students’ performance in one domain on their performance in the other, stresses the importance of the teachers’ role in teaching and enhancing both domains side by side.

Alongside the promising results of the current study, there are certain limitations that need to be considered. One main obstacle in this type of study is finding pure cognitive measures which do not depend on linguistic or numeric processing. The current study used measures which are related to numeric (RAN digits) and linguistic processing (Stroop). Specifically, Stroop color-word naming task which we used to measure inhibition involves reading of single words, and the Stroop interference task has been widely recognized as an indicator for reading automaticity (Logan, 1997). Therefore, the connection found between inhibition and reading fluency in first grade may be due to the task chosen and future studies should try to examine inhibition with a more neutral measure. Similarly, arithmetic fact fluency requires retrieval of the correct numerical answer and may be comparable to the speedy naming of digits in the RAN task. Hornung et al. (2017) found that RAN of numerical representations has a unique contribution to arithmetic. Therefore the connection found between RAN and mathematical fluency in first grade may also have been effected from the RAN task which was used. Although the tasks are not neutral it is important to note that they were selected according to the other studies in this field as mentioned in earlier sections. Future studies such include additional more neutral tasks in order to strengthen these results.

In addition, the participants of the current study included typical developing children in a relatively small sample size, and the collected data was analyzed through a cross-sectional approach. Therefore, the results of the present study may not be generalized to the entire population. Future studies should include a larger sample size, and also examine the suggested results among children with learning difficulties. Further research should continue to investigate the cross-domain connections between fluency in reading and arithmetic from a developmental perspective with a longitudinal study which will follow the same children and examine their development across the years.

Furthermore, the current examination focused on specific cognitive components which revealed only a certain portion of the explained variance of fluency performance. Additional examination of a wide range of cognitive components (e.g., cognitive flexibility, speed of processing, etc.) through tasks with different modalities (e.g., verbal and non-verbal processing) might broaden the presented findings concerning the predictive role of cognitive skills in the development of fluency. Also, a deep empirical investigation of the executed strategies in reading and arithmetic fluency is suggested as well.

## Conclusion

The results of the present study demonstrate that the cross-domain association between fluency in reading and arithmetic might be related to shared cognitive mechanisms. In addition, different patterns were found concerning the contribution of varied cognitive components (RAN, WM, and inhibition) to fluency in both academic domains. The observed trajectories might be related to the developmental nature of these abilities in the course of first through third grade. Therefore, it is important to be aware of that different cognitive abilities which contribute to the development of reading and mathematical fluency at different ages, these abilities should be strengthened among the children, to help to promote fluency in both domains.

Since fluency in reading and arithmetic are important for the development of more complex academic abilities in higher grades, the findings of the present study may have considerable educational implications. Specifically, understanding the cognitive foundations of both types of fluency might also assist with early identification of student’s having difficulties, and to develop effective instruction and interventions in order to improve fluency in both reading and arithmetic. In addition, the connection between reading and mathematical fluency may lead to influence of students’ performance in one domain on their performance in the other, and in fact stresses the importance of the teachers’ role in teaching and examining both domains side by side.

## Ethics Statement

This study was approved by the Institute of Education within the Ministry of Education in Israel as well as by the Research Ethics Committee of the Department of Education at the University of Haifa. Written consent was obtained from all the participants before the experiment.

## Author Contributions

RB and SS conceptualized this study and contributed to the writing and interpretation of the data. RB contributed to data collection and statistical analysis as well as to writing the first draft. Both authors have agreed to be accountable for the content of the work.

## Conflict of Interest Statement

The authors declare that the research was conducted in the absence of any commercial or financial relationships that could be construed as a potential conflict of interest.

## References

[B1] Adams J. W. Hitch G. J. (1997). Working memory and children’s mental addition. *J. Exp. Child Psychol.* 67 21–38. 9344485 10.1006/jecp.1997.2397

[B2] Araújo S. Reis A. Petersson K. M. Faísca L. (2015). Rapid automatized naming and reading performance: a meta-analysis. *J. Educ. Psychol.* 107:868. 10.1111/gbb.12299 27198479

[B3] Ashkenazi S. Black J. M. Abrams D. A. Hoeft F. Menon V. (2013). Neurobiological underpinnings of math and reading learning disabilities. *J. Learn. Disabil.* 46 549–569. 10.1177/0022219413483174 23572008 PMC3795983

[B4] Baddeley A. (1992). Working memory. *Science* 255 556–559. 1736359 10.1126/science.1736359

[B5] Baddeley A. (2003). Working memory: looking back and looking forward. *Nat. Rev. Neurosci.* 4:829. 10.1038/nrn1201 14523382

[B6] Barkley R. A. (1997). Behavioral inhibition, sustained attention, and executive functions: constructing a unifying theory of ADHD. *Psychol. Bull.* 121:65. 10.1037/0033-2909.121.1.65 9000892

[B7] Barrouillet P. Fayol M. Lathulière E. (1997). Selecting between competitors in multiplication tasks: an explanation of the errors produced by adolescents with learning difficulties. *Int. J. Behav. Dev.* 21 253–276.

[B8] Berg D. H. (2008). Working memory and arithmetic calculation in children: the contributory roles of processing speed, short-term memory, and reading. *J. Exp. Child Psychol.* 99 288–308. 10.1016/j.jecp.2007.12.002 18242631

[B9] Berninger V. W. Abbott R. D. Swanson H. L. Lovitt D. Trivedi P. Lin S. J. C. (2010). Relationship of word-and sentence-level working memory to reading and writing in second, fourth, and sixth grade. *Lang. Speech Hear. Serv. Sch.* 41 179–193. 10.1044/0161-1461(2009/08-0002) 19755637

[B10] Best J. R. Miller P. H. Naglieri J. A. (2011). Relations between executive function and academic achievement from ages 5 to 17 in a large, representative national sample. *Learn. Individ. Differ.* 21 327–336. 10.1016/j.lindif.2011.01.007 21845021 PMC3155246

[B11] Blair C. Razza R. P. (2007). Relating effortful control, executive function, and false belief understanding to emerging math and literacy ability in kindergarten. *Child Dev.* 78 647–663. 10.1111/j.1467-8624.2007.01019.x 17381795

[B12] Bowey J. A. (2005). “Predicting individual differences in learning to read,” *The Science of Reading: A Handbook*, eds Snowling M. J. Hulme C. (Hoboken, NJ: Wiley), 155–172. 10.1002/9780470757642.ch9

[B13] Brocki K. C. Eninger L. Thorell L. B. Bohlin G. (2010). Interrelations between executive function and symptoms of hyperactivity/impulsivity and inattention in preschoolers: a two year longitudinal study. *J. Abnorm. Child Psychol.* 38 163–171. 10.1007/s10802-009-9354-9 19763816

[B14] Brunswick N. Martin G. N. Rippon G. (2012). Early cognitive profiles of emergent readers: a longitudinal study. *J. Exp. Child Psychol.* 111 268–285. 10.1016/j.jecp.2011.08.001 21962459

[B15] Bull R. Scerif G. (2001). Executive functioning as a predictor of children’s mathematics ability: inhibition, switching, and working memory. *Dev. Neuropsychol.* 19 273–293. 10.1207/s15326942dn1903_3 11758669

[B16] Campbell J. I. (1990). Retrieval inhibition and interference in cognitive arithmetic. *Can. J. Psychol.* 44:445 10.1037/h0084266

[B17] Campbell J. I. D. Dowd R. R. Frick J. M. McCallum K. N. Metcalfe A. W. S. (2011). Neighborhood consistency and mem -ory for number facts. *Mem. Cognit.* 39 884–893. 10.3758/s13421-010-0064-x 21264638

[B18] Chambers F. (1997). What do we mean by fluency? *System* 25 535–544. 10.1016/s0346-251x(97)00046-8

[B19] Conners F. A. (2009). Attentional control and the simple view of reading. *Read. Writ.* 22 591–613. 10.1007/s11145-008-9126-x

[B20] Connor A. Franzen M. D. Sharp B. (1988). Effects of practice and differential instructions on Stroop performance. *Int. J. Clin. Neuropsychol.* 10 1–4.

[B21] Conway A. R. Engle R. W. (1994). Working memory and retrieval: a resource-dependent inhibition model. *J. Exp. Psychol.* 123:354. 10.1037//0096-3445.123.4.354 7996121

[B22] Cragg L. Gilmore C. (2014). Skills underlying mathematics: the role of executive function in the development of mathematics proficiency. *Trends Neurosci. Educ.* 3 63–68. 10.1016/j.tine.2013.12.001

[B23] Cragg L. Keeble S. Richardson S. Roome H. E. Gilmore C. (2017). Direct and indirect influences of executive functions on mathematics achievement. *Cognition* 162 12–26. 10.1016/j.cognition.2017.01.014 28189034

[B24] Crollen V. Noël M. P. (2015). The role of fingers in the development of counting and arithmetic skills. *Acta Psychol.* 156 37–44. 10.1016/j.actpsy.2015.01.007 25661746

[B25] Cui J. Georgiou G. K. Zhang Y. Li Y. Shu H. Zhou X. (2017). Examining the relationship between rapid automatized naming and arithmetic fluency in Chinese kindergarten children. *J. Exp. Child Psychol.* 154 146–163. 10.1016/j.jecp.2016.10.008 27883911

[B26] Cummine J. Aalto D. Ostevik A. Cheema K. Hodgetts W. (2018). “To name or not to name: that is the question”: the role of response inhibition in reading. *J. Psycholinguist. Res.* 47 999–1014. 10.1007/s10936-018-9572-9 29532285

[B27] de Hevia M. D. Vallar G. Girelli L. (2008). Visualizing numbers in the mind’s eye: The role of visuo-spatial processes in numerical abilities. *Neurosci. Biobehav. Rev.* 32 1361–1372. 10.1016/j.neubiorev.2008.05.015 18584868

[B28] De Smedt B. Janssen R. Bouwens K. Verschaffel L. Boets B. Ghesquière P. (2009). Working memory and individual differences in mathematics achievement: a longitudinal study from first grade to second grade. *J. Exp. Child Psychol.* 103 186–201. 10.1016/j.jecp.2009.01.004 19281998

[B29] Dempster F. N. (1992). The rise and fall of the inhibitory mechanism: toward a unified theory of cognitive development and aging. *Dev. Rev.* 12 45–75. 10.1016/0273-2297(92)90003-k

[B30] Dickinson D. K. Porche M. V. (2011). Relation between language experiences in preschool classrooms and children’s kindergarten and fourth-grade language and reading abilities. *Child Dev.* 82 870–886. 10.1111/j.1467-8624.2011.01576.x 21413936

[B31] Dowsett S. M. Livesey D. J. (2000). The development of inhibitory control in preschool children: effects of “executive skills” training. *Dev. Psychobiol.* 36 161–174. 10.1002/(sici)1098-2302(200003)36:2<161::aid-dev7>3.0.co;2-0 10689286

[B32] Durand M. Hulme C. Larkin R. Snowling M. (2005). The cognitive foundations of reading and arithmetic skills in 7-to 10-year-olds. *J. Exp. Child Psychol.* 91 113–136. 10.1016/j.jecp.2005.01.003 15890173

[B33] Espy K. A. McDiarmid M. M. Cwik M. F. Stalets M. M. Hamby A. Senn T. E. (2004). The contribution of executive functions to emergent mathematic skills in preschool children. *Dev. Neuropsychol.* 26 465–486. 10.1207/s15326942dn2601_6 15276905

[B34] Friso-van den Bos I. van der Ven S. H. Kroesbergen E. H. van Luit J. E. (2013). Working memory and mathematics in primary school children: a meta-analysis. *Educ. Res. Rev.* 10 29–44. 10.1016/j.edurev.2013.05.003

[B35] Fuchs L. S. Fuchs D. Compton D. L. Powell S. R. Seethaler P. M. Capizzi A. M. (2006). The cognitive correlates of third-grade skill in arithmetic, algorithmic computation, and arithmetic word problems. *J. Educ. Psychol.* 98:29 10.1037/0022-0663.98.1.29

[B36] Fuchs L. S. Fuchs D. Hosp M. K. Jenkins J. R. (2001). Oral reading fluency as an indicator of reading competence: a theoretical, empirical, and historical analysis. *Sci. Stud. Read.* 5 239–256. 10.4324/9781410608246-3

[B37] Fuchs L. S. Geary D. C. Fuchs D. Compton D. L. Hamlett C. L. (2016). Pathways to third-grade calculation versus word-reading competence: are they more alike or different?. *Child Dev.* 87 558–567. 10.1111/cdev.12474 26700885 PMC4809764

[B38] Gathercole S. E. Baddeley A. D. (1993). Phonological working memory: a critical building block for reading development and vocabulary acquisition? *Eur. J. Psychol. Educ.* 8:259 10.1007/bf03174081

[B39] Gazzaley A. Cooney J. W. Rissman J. D’esposito M. (2005). Top-down suppression deficit underlies working memory impairment in normal aging. *Nat. Neurosci.* 8:1298. 10.1038/nn1543 16158065

[B40] Geary D. C. Brown S. C. Samaranayake V. A. (1991). Cognitive addition: a short longitudinal study of strategy choice and speed-of-processing differences in normal and mathematically disabled children. *Dev. Psychol.* 27:787 10.1037/0012-1649.27.5.787

[B41] Geary D. C. Hoard M. K. Byrd-Craven J. DeSoto M. C. (2004). Strategy choices in simple and complex addition: contributions of working memory and counting knowledge for children with mathematical disability. *J. Exp. Child Psychol.* 88 121–151. 10.1016/s0022-0965(04)00033-5 15157755

[B42] Georgiou G. K. Das J. P. Hayward D. V. (2008a). Comparing the contribution of two tests of working memory to reading in relation to phonological awareness and rapid naming speed. *J. Res. Read.* 31 302–318. 10.1111/j.1467-9817.2008.00373.x

[B43] Georgiou G. K. Parrila R. Papadopoulos T. C. (2008b). Predictors of word decoding and reading fluency across languages varying in orthographic consistency. *J. Educ. Psychol.* 100:566 10.1037/0022-0663.100.3.566

[B44] Georgiou G. K. Parrila R. Kirby J. R. (2009). RAN components and reading development from grade 3 to grade 5: what underlies their relationship? *Sci. Stud. Read.* 13 508–534. 10.1080/10888430903034796

[B45] Georgiou G. K. Torppa M. Manolitsis G. Lyytinen H. Parrila R. (2012). Longitudinal predictors of reading and spelling across languages varying in orthographic consistency. *Read. Writ.* 25 321–346. 10.1007/s11145-010-9271-x

[B46] Georgiou G. K. Parrila R. Cui Y. Papadopoulos T. C. (2013a). Why is rapid automatized naming related to reading? *J. Exp. Child Psychol.* 115 218–225. 10.1016/j.jecp.2012.10.015 23384823

[B47] Georgiou G. K. Tziraki N. Manolitsis G. Fella A. (2013b). Is rapid automatized naming related to reading and mathematics for the same reason (s)? A follow-up study from kindergarten to Grade 1. *J. Exp. Child Psychol.* 115 481–496. 10.1016/j.jecp.2013.01.004 23506806

[B48] Georgiou G. K. Manolitsis G. Tziraki N. (2015). “Is intelligence relevant in reading “μáνα” and in calculating “3+5”?,” in *Cognition Intelligence and Achievement* eds Parrila R. K. Kirby J. R. Papadopoulos T. (Cambridge, MA: Academic Press), 225–243 10.1016/b978-0-12-410388-7.00012-9

[B49] Gilmore C. Keeble S. Richardson S. Cragg L. (2015). The role of cognitive inhibition in different components of arithmetic. *Zdm* 47 771–782. 10.1016/j.jecp.2019.01.012 30807905

[B50] Graf P. Uttl B. Tuokko H. (1995). Color-and picture-word Stroop tests: performance changes in old age. *J. Clin. Exp. Neuropsychol.* 17 390–415. 10.1080/01688639508405132 7650102

[B51] Granvald V. Marciszko C. (2016). Relations between key executive functions and aggression in childhood. *Child Neuropsychol.* 22 537–555. 10.1080/09297049.2015.1018152 25833167

[B52] Hart S. A. Petrill S. A. Thompson L. A. (2010). A factorial analysis of timed and untimed measures of mathematics and reading abilities in school aged twins. *Learn. Individ. Differ.* 20 63–69. 10.1016/j.lindif.2009.10.004 20161680 PMC2821061

[B53] Hart S. A. Petrill S. A. Thompson L. A. Plomin R. (2009). The ABCs of math: a genetic analysis of mathematics and its links with reading ability and general cognitive ability. *J. Educ. Psychol.* 101:388. 10.1037/a0015115 20157630 PMC2821062

[B54] Hasher L. Zacks R. T. (1988). “Working memory, comprehension, and aging: a review and a new view,” in *Psychology of Learning And Motivation*, ed. Federmeier K. D. Vol. 22 (Cambridge, MA: Academic Press), 193–225 10.1016/s0079-7421(08)60041-9

[B55] Hasher L. Zacks R. T. May C. P. (1999). “Inhibitory control, circadian arousal, and age,” in *Attention and performance. Attention and performance XVII: Cognitive regulation of performance: Interaction of theory and application*, eds Gopher D. Koriat A. (Cambridge, MA: The MIT Press), 653–675.

[B56] Hecht S. A. (2002). Counting on working memory in simple arithmetic when counting is used for problem solving. *Mem. Cogn.* 30 447–455. 10.3758/bf03194945 12061765

[B57] Hornung C. Martin R. Fayol M. (2017). General and specific contributions of RAN to reading and arithmetic fluency in first graders: a longitudinal latent variable approach. *Front. Psychol.* 8:1746. 10.3389/fpsyg.2017.01746 29056920 PMC5635811

[B58] Hoshi Y. Oda I. Wada Y. Ito Y. Yamashita Y. Oda M. (2000). Visuospatial imagery is a fruitful strategy for the digit span backward task: a study with near-infrared optical tomography. *Cogn. Brain Res.* 9 339–342. 10.1016/s0926-6410(00)00006-9 10808144

[B59] Hudson R. F. Lane H. B. Pullen P. C. (2005). Reading fluency assessment and instruction: what, why, and how? *Read. Teach.* 58 702–714. 10.1598/rt.58.8.1

[B60] Jacobson L. A. Ryan M. Martin R. B. Ewen J. Mostofsky S. H. Denckla M. B. (2011). Working memory influences processing speed and reading fluency in ADHD. *Child Neuropsychol.* 17 209–224. 10.1080/09297049.2010.532204 21287422 PMC3309419

[B61] Jordan N. C. Hanich L. B. Kaplan D. (2003). A longitudinal study of mathematical competencies in children with specific mathematics difficulties versus children with comorbid mathematics and reading difficulties. *Child Dev.* 74 834–850. 10.1111/1467-8624.00571 12795393 PMC2791887

[B62] Katzir T. Schiff R. Kim Y. S. (2012). The effects of orthographic consistency on reading development: a within and between cross-linguistic study of fluency and accuracy among fourth grade English-and Hebrew-speaking children. *Learn. Individ. Differ.* 22 673–679. 10.1016/j.lindif.2012.07.002

[B63] Kaufmann L. Lochy A. Drexler A. Semenza C. (2004). Deficient arithmetic fact retrieval—storage or access problem?: a case study. *Neuropsychologia* 42 482–496. 10.1016/j.neuropsychologia.2003.09.004 14728921

[B64] Kim Y. S. Petscher Y. Schatschneider C. Foorman B. (2010). Does growth rate in oral reading fluency matter in predicting reading comprehension achievement? *J. Educ. Psychol.* 102:652 10.1037/a0019643

[B65] Klauda S. L. Guthrie J. T. (2008). Relationships of three components of reading fluency to reading comprehension. *J. Educ. Psychol.* 100:310 10.1037/0022-0663.100.2.310

[B66] Koponen T. Aunola K. Ahonen T. Nurmi J. E. (2007). Cognitive predictors of single-digit and procedural calculation skills and their covariation with reading skill. *J. Exp. Child Psychol.* 97 220–241. 10.1016/j.jecp.2007.03.001 17560969

[B67] Koponen T. Georgiou G. Salmi P. Leskinen M. Aro M. (2017). A meta-analysis of the relation between RAN and mathematics. *J. Educ. Psychol.* 109:977 10.1037/edu0000182

[B68] Koponen T. Salmi P. Eklund K. Aro T. (2013). Counting and RAN: predictors of arithmetic calculation and reading fluency. *J. Educ. Psychol.* 105:162 10.1037/a0029285

[B69] Koponen T. Salmi P. Torppa M. Eklund K. Aro T. Aro M. (2016). Counting and rapid naming predict the fluency of arithmetic and reading skills. *Contemp. Educ. Psychol.* 44 83–94. 10.1016/j.cedpsych.2016.02.004

[B70] Korpipää H. Koponen T. Aro M. Tolvanen A. Aunola K. Poikkeus A. M. (2017). Covariation between reading and arithmetic skills from Grade 1 to Grade 7. *Contemp. Educ. Psychol.* 51 131–140. 10.1016/j.cedpsych.2017.06.005

[B71] Kulak A. G. (1993). Parallels between math and reading disability: common issues and approaches. *J. Learn. Disabil.* 26 666–673. 10.1177/002221949302601004 8151206

[B72] LaBerge D. Samuels S. J. (1974). Toward a theory of automatic information processing in reading. *Cogn. Psychol.* 6 293–323. 10.1016/0010-0285(74)90015-2

[B73] Landerl K. Wimmer H. (2008). Development of word reading fluency and spelling in a consistent orthography: an 8-year follow-up. *J. Educ. Psychol.* 100:150 10.1037/0022-0663.100.1.150

[B74] Laski E. V. Casey B. M. Yu Q. Dulaney A. Heyman M. Dearing E. (2013). Spatial skills as a predictor of first grade girls’ use of higher level arithmetic strategies. *Learn. Individ. Differ.* 23 123–130. 10.1016/j.lindif.2012.08.001

[B75] LeFevre J. A. Berrigan L. Vendetti C. Kamawar D. Bisanz J. Skwarchuk S. L. (2013). The role of executive attention in the acquisition of mathematical skills for children in Grades 2 through 4. *J. Exp. Child Psychol.* 114 243–261. 10.1016/j.jecp.2012.10.005 23168083

[B76] Leon-Carrion J. García-Orza J. Pérez-Santamaría F. J. (2004). Development of the inhibitory component of the executive functions in children and adolescents. *Int. J. Neurosci.* 114 1291–1311. 10.1080/00207450490476066 15370187

[B77] Lipka O. Katzir T. Shaul S. (2016). “The basis of reading fluency in first grade of hebrew speaking children,” in *Reading Fluency* eds A Khateb, and I Bar. (Cham: Springer), 91–104 10.1007/978-3-319-30478-6_6

[B78] Logan G. D. (1988). Toward an instance theory of automatization. *Psychol. Rev.* 95:492 10.1037//0033-295x.95.4.492

[B79] Logan G. D. (1997). Automaticity and reading: Perspectives from the instance theory of automatization. *Read. Writ. Q.* 13 123–146. 10.1080/1057356970130203

[B80] Mabbott D. J. Bisanz J. (2003). Developmental change and individual differences in children’s multiplication. *Child Dev.* 74 1091–1107. 10.1111/1467-8624.00594 12938706

[B81] Mammarella I. C. Bomba M. Caviola S. Broggi F. Neri F. Lucangeli D. (2013). Mathematical difficulties in nonverbal learning disability or co-morbid dyscalculia and dyslexia. *Dev. Neuropsychol.* 38 418–432. 10.1080/87565641.2013.817583 23971493

[B82] Mazzocco M. M. Grimm K. J. (2013). Growth in rapid automatized naming from grades K to 8 in children with math or reading disabilities. *J. Learn. Disabil.* 46 517–533. 10.1177/0022219413477475 23449728 PMC5206966

[B83] McInerney R. J. Hrabok M. Kerns K. A. (2005). The children’s size-ordering task: a new measure of nonverbal working memory. *J. Clin. Exp. Neuropsychol.* 27 735–745. 10.1081/13803390490918633 16019649

[B84] Meyer M. L. Salimpoor V. N. Wu S. S. Geary D. C. Menon V. (2010). Differential contribution of specific working memory components to mathematics achievement in 2nd and 3rd graders. *Learn. Individ. Differ.* 20 101–109. 10.1016/j.lindif.2009.08.004 21660238 PMC3109434

[B85] Meyer M. S. Felton R. H. (1999). Repeated reading to enhance fluency: old approaches and new directions. *Ann. Dyslexia* 49 283–306. 10.1007/s11881-999-0027-8

[B86] Meyer M. S. Wood F. B. Hart L. A. Felton R. H. (1998). Selective predictive value of rapid automatized naming in poor readers. *J. Learn. Disabil.* 31 106–117. 10.1177/002221949803100201 9529781

[B87] Miller M. R. Giesbrecht G. F. Müller U. McInerney R. J. Kerns K. A. (2012). A latent variable approach to determining the structure of executive function in preschool children. *J. Cogn. Dev.* 13 395–423. 10.1080/15248372.2011.585478

[B88] Miyake A. Friedman N. P. Emerson M. J. Witzki A. H. Howerter A. Wager T. D. (2000). The unity and diversity of executive functions and their contributions to complex “frontal lobe” tasks: a latent variable analysis. *Cogn. Psychol.* 41 49–100. 10.1006/cogp.1999.0734 10945922

[B89] Monette S. Bigras M. Guay M. C. (2011). The role of the executive functions in school achievement at the end of Grade 1. *J. Exp. Child Psychol.* 109 158–173. 10.1016/j.jecp.2011.01.008 21349537

[B90] National Mathematics Advisory Panel (2008). *Foundations For Success: The Final Report of The National Mathematics Advisory Panel*. Washington, DC: US Department of Education

[B91] Nevo E. Bar-Kochva I. (2015). The relations between early working memory abilities and later developing reading skills: a longitudinal study from kindergarten to fifth grade. *Mind Brain Educ.* 9 154–163. 10.1111/mbe.12084

[B92] Noreen S. MacLeod M. D. (2015). What do we really know about cognitive inhibition? Task demands and inhibitory effects across a range of memory and behavioural tasks. *PLoS One* 10 e0134951. 10.1371/journal.pone.0134951 26270470 PMC4536050

[B93] Nunes T. Bryant P. Barros R. Sylva K. (2012). The relative importance of two different mathematical abilities to mathematical achievement. *Br. J. Educ. Psychol.* 82 136–156. 10.1111/j.2044-8279.2011.02033.x 22429062

[B94] Office of Pedagogical Affairs Israel Ministry of Education (2006). *Math Curriculum for Elementary Schools in Israel*. Jerusalem: Israel Ministry of Education

[B95] Petrill S. Logan J. Hart S. Vincent P. Thompson L. Kovas Y. (2012). Math fluency is etiologically distinct from untimed math performance, decoding fluency, and untimed reading performance: evidence from a twin study. *J. Learn. Disabil.* 45 371–381. 10.1177/0022219411407926 21890908 PMC3413280

[B96] Price G. R. Mazzocco M. M. Ansari D. (2013). Why mental arithmetic counts: brain activation during single digit arithmetic predicts high school math scores. *J. Neurosci.* 33 156–163. 10.1523/JNEUROSCI.2936-12.2013 23283330 PMC6618631

[B97] Pritchard V. E. Neumann E. (2004). Negative priming effects in children engaged in nonspatial tasks: evidence for early development of an intact inhibitory mechanism. *Dev. Psychol.* 40:191. 10.1037/0012-1649.40.2.191 14979760

[B98] Purpura D. J. Schmitt S. A. (2018). Cross-domain development of early academic and cognitive skills. *Early Child. Res. Q.* 46 1–4 10.1016/j.ecresq.2018.10.009

[B99] Qin S. Cho S. Chen T. Rosenberg-Lee M. Geary D. C. Menon V. (2014). Hippocampal-neocortical functional reorganization underlies children’s cognitive development. *Nat. Neurosci.* 17:1263. 10.1038/nn.3788 25129076 PMC4286364

[B100] Rohl M. Pratt C. (1995). Phonological awareness, verbal working memory and the acquisition of literacy. *Read. Writ.* 7 327–360. 10.1007/bf01027723

[B101] Ruddy J. Ciancio D. Skinner C. H. Blonder M. (2018). Receiver operating characteristic analysis of oral reading fluency predicting broad reading scores. *Contemp. Sch. Psychol.* 6 1–13.

[B102] Sacks T. L. Clark C. R. Pols R. G. Geffen L. B. (1991). Comparability and stability of performance of six alternate forms of the Dodrill-Stroop Colour-Word Test. *Clin. Neuropsychol.* 5 220–225. 10.1080/13854049108404093

[B103] Saiegh-Haddad E. (2005). Correlates of reading fluency in Arabic: diglossic and orthographic factors. *Read. Writ.* 18 559–582. 10.1007/s11145-005-3180-4

[B104] Schadler M. Thissen D. M. (1981). The development of automatic word recognition and reading skill. *Mem. Cogn.* 9 132–141. 10.3758/bf03202327 7242326

[B105] Schiff R. Kahta S. Katzir T. (2006). *Single-Word Reading Test: Vowelized and Unvowelized Word Reading*. Ramat Gan: Haddad Center, Bar-Ilan University.

[B106] Schwanenflugel P. J. Meisinger E. B. Wisenbaker J. M. Kuhn M. R. Strauss G. P. Morris R. D. (2006). Becoming a fluent and automatic reader in the early elementary school years. *Read. Res. Q.*, 41 496–522. 10.1598/rrq.41.4.4 20072665 PMC2805254

[B107] Seitz K. Schumann-Hengsteler R. (2002). Phonological loop and central executive processes in mental addition and multiplication. *Psychol. Test Assess. Model.* 44:275.

[B108] Senn T. E. Espy K. A. Kaufmann P. M. (2004). Using path analysis to understand executive function organization in preschool children. *Dev. Neuropsychol.* 26 445–464. 10.1207/s15326942dn2601_5 15276904

[B109] Shany M. Share D. L. (2011). Subtypes of reading disability in a shallow orthography: a double dissociation between accuracy-disabled and rate-disabled readers of Hebrew. *Ann. Dyslexia* 61 64–84. 10.1007/s11881-010-0047-4 21108026

[B110] Shany M. Lachman D. Shalem Z. Bahat A. Zeiger T. (2006). *Aleph-Taph a Test for the Diagnosis of Reading and Writing Disabilities, Based on National Israeli Norms*. Tel Aviv: Yesod Publishing.

[B111] Share D. L. (1995). Phonological recoding and self-teaching: sine qua non of reading acquisition. *Cognition* 55 151–218. 10.1016/0010-0277(94)00645-2 7789090

[B112] Share D. L. Bar-On A. (2017). Learning to read a semitic abjad: the triplex model of hebrew reading development. *J. Learn. Disabil.* 51 444–453. 10.1177/0022219417718198 28703637

[B113] Shechter A. Lipka O. Katzir T. (2018). Predictive models of word reading fluency in hebrew. *Front. Psychol.* 9:1882. 10.3389/fpsyg.2018.01882 30356726 PMC6189333

[B114] Simmons F. R. Singleton C. (2008). Do weak phonological representations impact on arithmetic development? A review of research into arithmetic and dyslexia. *Dyslexia* 14 77–94. 10.1002/dys.341 17659647

[B115] Singer V. Strasser K. (2017). The association between arithmetic and reading performance in school: a meta-analytic study. *Sch. Psychol. Q.* 32:435. 10.1037/spq0000197 28221052

[B116] Soltanlou M. Pixner S. Nuerk H. C. (2015). Contribution of working memory in multiplication fact network in children may shift from verbal to visuo-spatial: a longitudinal investigation. *Front. Psychol.* 6:1062. 10.3389/fpsyg.2015.01062 26257701 PMC4512035

[B117] Stanovich K. E. Cunningham A. E. West R. F. (1981). A longitudinal study of the development of automatic recognition skills in first graders. *J. Read. Behav.* 13 57–74. 10.1080/10862968109547394

[B118] Stroop J. R. (1935). Studies of interference in serial verbal reactions. *J. Exp. Psychol.* 18:643 10.1037/h0054651

[B119] Swanson H. L. Jerman O. (2007). The influence of working memory on reading growth in subgroups of children with reading disabilities. *J. Exp. Child Psychol.* 96 249–283. 10.1016/j.jecp.2006.12.004 17437762

[B120] Swanson L. Kim K. (2007). Working memory, short-term memory, and naming speed as predictors of children’s mathematical performance. *Intelligence* 35 151–168. 10.1016/j.intell.2006.07.001

[B121] Torgesen J. K. Rashotte C. A. Wagner R. K. (1999). *Towre: Test of Word Reading Efficiency*. Austin, TX: Pro-ed.

[B122] Van der Sluis S. de Jong P. F. van der Leij A. (2007). Executive functioning in children, and its relations with reasoning, reading, and arithmetic. *Intelligence* 35 427–449. 10.1016/j.intell.2006.09.001

[B123] Vanbinst K. Ceulemans E. Ghesquière P. De Smedt B. (2015). Profiles of children’s arithmetic fact development: a model-based clustering approach. *J. Exp. Child Psychol.* 133 29–46. 10.1016/j.jecp.2015.01.003 25731679

[B124] Von Aster M. G. Shalev R. S. (2007). Number development and developmental dyscalculia. *Dev. Med. Child Neurol.* 49 868–873. 10.1111/j.1469-8749.2007.00868.x 17979867

[B125] Vukovic R. K. Lesaux N. K. Siegel L. S. (2010). The mathematics skills of children with reading difficulties. *Learn. Individ. Differ.* 20 639–643. 10.1016/j.lindif.2010.08.004

[B126] Warmington M. Hulme C. (2012). Phoneme awareness, visual-verbal paired-associate learning, and rapid automatized naming as predictors of individual differences in reading ability. *Sci. Stud. Read.* 16 45–62. 10.1080/10888438.2010.534832

[B127] Wechsler D. (1991). *Wisc-III: Wechsler Intelligence Scale For Children: Manual*. Agra: Psychological Corporation

[B128] Wolf M. Katzir-Cohen T. (2001). Reading fluency and its intervention. *Sci. Stud. Read.* 5 211–239. 10.1207/s1532799xssr0503_2

[B129] Woodcock R. W. McGrew K. S. Mather N. Schrank F. (2001). *Woodcock-Johnson R III NU Tests of Achievement*. Itasca, IL: Riverside

[B130] Yeniad N. Malda M. Mesman J. van IJzendoorn M. H. Pieper S. (2013). Shifting ability predicts math and reading performance in children: a meta-analytical study. *Learn. Individ. Differ.* 23 1–9. 10.1016/j.lindif.2012.10.004

[B131] Zaunmüller L. Domahs F. Dressel K. Lonnemann J. Klein E. Ischebeck A. (2009). Rehabilitation of arithmetic fact retrieval via extensive practice: a combined fMRI and behavioural case-study. *Neuropsychol. Rehabil.* 19 422–443. 10.1080/09602010802296378 18949656

[B132] Zelazo P. D. Müller U. (2002). “Executive function in typical and atypical development,” in *Blackwell Handbook of Childhood Cognitive Development*, ed. Goswami U. (Malden, MA: Blackwell Publishing), 445–469. 10.1002/9780470996652.ch20

